# A breeding strategy for improving drought and salt tolerance of poplar based on CRISPR/Cas9

**DOI:** 10.1111/pbi.14147

**Published:** 2023-08-03

**Authors:** Tengqian Zhang, Weixi Zhang, Changjun Ding, Zanmin Hu, Chengming Fan, Jing Zhang, Zhenghong Li, Songfeng Diao, Le Shen, Bingyu Zhang, Guifeng Liu, Xiaohua Su

**Affiliations:** ^1^ State Key Laboratory of Tree Genetics and Breeding, Research Institute of Forestry Chinese Academy of Forestry, Northeast Forestry University Harbin China; ^2^ Key Laboratory of Tree Breeding and Cultivation of State Forestry Administration, Research Institute of Forestry Chinese Academy of Forestry Beijing China; ^3^ Institute of Genetics and Developmental Biology Chinese Academy of Sciences Beijing China; ^4^ Co‐Innovation Center for Sustainable Forestry in Southern China Nanjing Forestry University Nanjing China

**Keywords:** CRISPR/Cas9, poplar, HyPRP1, salt stress, drought stress, ROS, ion flux

Afforestation and vegetation restoration are challenging tasks in arid, semi‐arid and saline‐alkali areas. It is now highly demanded in agroforestry to generate new stress‐resistant trees that adapt to these difficult environments. As a high‐efficiency, strong precision and versatility tool, the CRISPR/Cas9 technology has been utilized broadly in major crops and model plants (Li *et al*., 2022; Zhang *et al*., 2021). However, its application in trees has rarely been reported, especially in drought and salt‐tolerance fields. Hybrid proline‐rich proteins are proline and hydroxyproline‐rich cell wall structural proteins and can participate in biotic and abiotic stress responses as negative regulatory factors (Banday *et al*., 2022). In this study, we identified *PagHyPRP1* (*PagHyPRP1A* and *PagHyPRP1B*) as potential negative regulators of drought and salt stress response, which were the targets for gene editing using an important cultivated variety *Populus alba* × *P*. *glandulosa* (Figure 1a; Figure S1). *PagHyPRP1* was found to be mainly and highly expressed in the roots, which is different from the previous reports that showed dominant expression in the leaves of *Poncirus trifoliata* and tomato (Saikia *et al*., 2020; Figure S1). Two *PagHyPRP1* specific sgRNAs without potential off‐target sites were identified and constructed into the pHZM58 vector, which was used for *Agrobacterium*‐mediated transformation (Figure 1b,c). Nine mutants were obtained, 60% of total transformants, including two homozygous, two allelic and five chimeric mutant lines. All mutants resulted from frameshift caused by 1 bp insertion or 1–4 bp deletions. Four mutants (*prp‐1*, *prp‐2*, *prp‐4*, and *prp‐6*) validated after asexual propagation were selected for further characterization on drought and salt tolerance (Figure 1d–f). The mutants showed significantly decreased expression of *PagHyPRP1* in the roots and leaves under normal and stress conditions, compared with wildtype (WT) and overexpression lines (OEs; Figure 1g).

**Figure 1:**
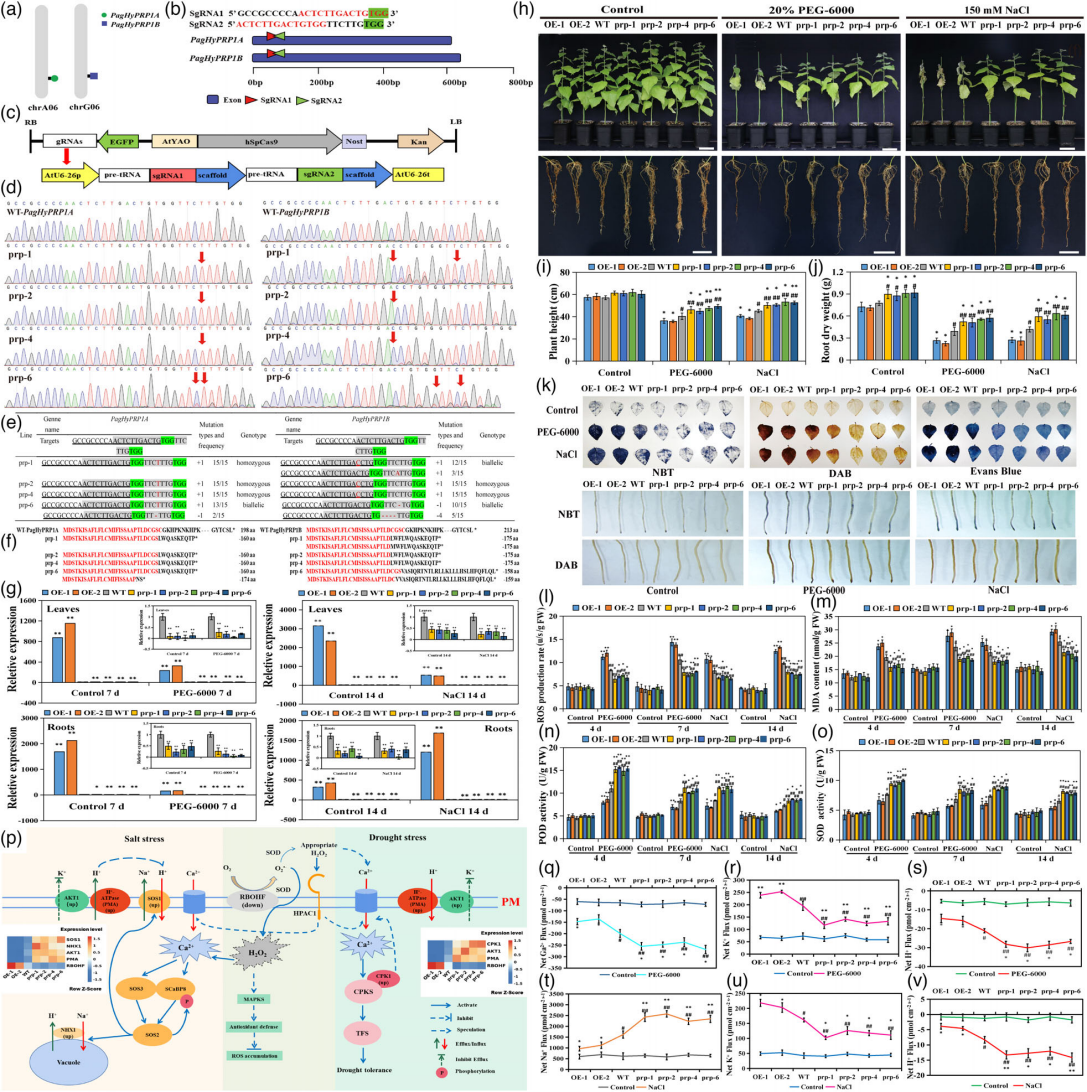
CRISPR/Cas9‐mediated mutations of *PagHyPRP1* improve drought and salt tolerance in poplar. (a) The chromosome loci of *PagHyPRP1A* and *PagHyPRP1B* on the poplar genome. (b) The two designed sgRNAs. Red letters are overlapping regions between the two paralogs. (c)The constructs used in this study. (d, e) Sequencing chromatograms and corresponding genetic analysis of target sites in mutants. (f) Multiple sequence alignment of the predicted PagHyPRP1 motifs of WT and mutants. (g) RT‐qPCR analysis of *PagHyPRP1* in leaves and roots of mutants, OEs and WT upon the stress treatments. (h) Phenotypic analysis of mutants, OEs and WT upon the stress treatment. Bars = 10 cm. (i, j) Plant height and root dry weight of mutants, OEs and WT upon the stress treatments. (k) NBT, DAB and Evans blue staining of mutants after 3 h of treatment. (l–o) ROS concentrations, MDA contents, POD and SOD activities in mutants, OEs and WT in response to the stress treatments. (p) ROS signalling pathway analysis of mutant lines in response to drought and salt stress. The relative expression levels in *p* were Z‐scaled log_2_FPKM. (q–v) Ca^2+^, Na^+^, K^+^ and H^+^ ion flux of root tips in the mutants, OEs and WT upon the stress treatments. The chemical staining test was done with the concentration of 10% PEG‐6000 and 150 mm NaCl, and the other tests were done with 20% PEG‐6000 and 150 mm NaCl. Data = means ± SD from three or six biological replicates. *, ** indicates significant differences from WT (*P < 0.05; **P < 0.01). ^#^, ^##^ indicates significant differences from OEs (^#^ *P* < 0.05; ^##^ *P* < 0.01).

Healthy plants with consistent growth were selected for simulated soil drought (20% PEG‐6000) and salt (150 mm NaCl) stress tests (Figure S2). After 21 days without stress treatment, the mutants displayed significantly better growth than OEs and WT, including plant height, root length and dry weight of stem and roots. There was no significant difference observed between OEs and WT (Figure 1h). Under the PEG‐6000 and NaCl treatments, four mutants maintained normal growth of the middle and upper leaves. In contrast, most of the leaves in WT and OE lines experienced wilting, withering, and even premature abscission (Figure 1h). Compared to WT, the mutants showed significantly higher height (46.97 and 51.69 cm), root dry weight (0.54 and 0.60 g), stem diameter (3.84 and 4.14 cm), stem dry weight (1.15 and 1.29 g), and root‐shoot ratio (0.17 and 0.18), respectively. On the contrary, the OEs showed significantly lower growth by 10.16%–42.74% and 10.05%–37.60% compared to WT (Figure 1i,j; Figure S3). Consistently, we observed similar responses from the treatment done with *in vitro* plants with 3% PEG‐6000 and 75, 100 mm NaCl stress tests (Figure S4). These results suggested that the mutation of *PagHyPRP1* could enhance poplar trees' growth and root development, and these *PagHyPRP1*mutants could greatly contribute to the poplar germplasm with improved drought and salt tolerance.

To further evaluate the tolerance to drought and salt stress, we measured oxidative damage level, antioxidant and osmoregulation capacity. Compared to normal conditions, all plants showed deepened NBT, DAB and Evans blue staining under drought and salt stress treatments. However, the mutants consistently exhibited the lightest staining, followed by WT, while the deepest colour in the OEs (Figure 1k). The ROS concentrations, ${O_{2}}^{\cdot -}$, H_2_O_2_ and MDA contents increased in all lines during the extension of both stresses. Compared to WT, the mutants showed significantly lower levels of ROS, ${O_{2}}^{\cdot -}$, H_2_O_2_, and MDA (7.69 and 7.64 u/s/g; 137.01 and 119.06 nmol/g; 17.70 and 19.35 μmol/g; 19.17 and 21.01 nmol/g) by 14.63%–31.71% and 13.78%–27.79% after 7 days PEG‐6000 and 14 days salt treatment, respectively. However, their counterparts in OEs were increased to 1.13–1.36 times and 1.12–1.35 times (Figure 1l,m; Figure S5). Furthermore, the proline content, POD and SOD activities peaked 4 days (PEG‐6000) and 7 days (salt) after the treatments. At the same time, the mutants displayed significantly enhanced responses with 11.97%–43.07% and 13.27%–37.44% more compared to WT, while the OEs significantly reduced about 13.23%–28.04% and 10.60%–20.62%, respectively (Figure 1n,o; Figure S6). These findings indicated that mutants could enhance antioxidant and osmoregulation abilities by increasing POD and SOD peroxidase activities and proline content, and reduce oxidative damage through decreasing ${O_{2}}^{\cdot -}$, H_2_O_2_, ROS and MDA contents, which leads to improved drought and salt tolerance.

To investigate the ROS signalling alteration of the mutant in response to drought and salt stress, we measured the key ion flux and analysed related gene expression in roots. As shown in Figure 1p, lower expression of *RBOHF* in mutants could modulate appropriate ROS signalling, such as H_2_O_2_, then activate various Ca^2+^ channels and promote Ca^2+^ influx in extracellular via HPCA1. Under drought‐simulated stress, Ca^2+^ signalling could activate *CPK1* and other CPKS to promote Ca^2+^ signal transduction and trigger transcriptional responses to stress. Meanwhile, the higher levels of *CPK1* might, in turn, promote Ca^2+^ influx (1.19–1.35 times of WT) (Chen *et al*., 2021; Mittler *et al*., 2022; Zhang *et al*., 2022). In addition, the increased H^+^ influx (1.27–1.43 times of WT) and decreased K^+^ efflux (0.60–0.72 times of WT) might result from the upregulation of *PMA* and *AKT*. Higher levels of ion concentration in the mutants could benefit the formation of osmoregulation substances and reduce the intracellular water potential, which then improves the drought tolerance by maintaining the balance of osmotic pressure (Figure 1q–s). Likewise, under salt stress, the appropriate ROS might form a ‘ROS‐Ca^2+^’ hub to activate the SOS signalling pathway (Chen *et al*., 2021). Hence the upregulated expression of *SOS1* and *NHX1* in the mutants could activate Na^+^/H^+^ transporters to promote Na^+^ efflux (1.38–1.59 times of WT) and H^+^ influx (1.45–1.70 times of WT). Besides, the increased levels of *PMA* and *AKT1* expression in the mutants indicated limit levels of K^+^ efflux (0.63–0.78 times of WT) and enhanced electrogenic proton (H^+^) pumps, resulting in higher levels of salt tolerance in the mutants by maintaining the balance of Na^+^/K^+^ in intracellular (Figure 1t–v).

In conclusion, this study shows a successful multilocus genome editing on *PagHyPRP1* of *P. alba* × *P. glandulosa* by CRISPR/Cas9, with a 60% editing efficiency. The mutations were stable over the asexual propagation procedure, and the *PagHyPRP1* expression was almost undetectable. The four new mutants were integrated into the poplar germplasms with significantly improved drought and salt tolerance. This work also opens a new avenue for applying CRISPR/Cas9 to create new germplasm for stress resistance in forest trees. This great input to the new poplar germplasms can facilitate forestation in difficult environments.

## Conflict of interest

The authors declare no conflicts of interest.

## Author contribution

X.S. and C.D. designed the research. T.Z., W.Z., C.D., H.Z., F.C., J.Z., Z.L., S.D. and L.S. performed the experiments and data analysis. T.Z., W.Z., C.D., B.Z. and G.L. wrote and revised the manuscript. All authors read and approved the manuscript.

## Supporting information


**Figure S1** Multiple alignment, phylogenetic analysis and expression analysis of *PagHyPRP1A* and *PagHyPRP1B*.
**Figure S2** Healthy plants with consistent growth were selected for simulated soil drought (20% PEG‐6000) and salt (150 mM NaCl) stress tests.
**Figure S3** Stem diameter, stem dry weight and root‐shoot ratio of mutant lines, OE lines and the WT under drought and salt stress.
**Figure S4** Phenotypes analysis of mutant lines, OEs, and WT upon the stress treatment *in vitro* for 30 days.
**Figure S5** The contents of ${O_{2}}^{\cdot -}$ and H_2_O_2_ in mutants, OEs and WT upon the drought and salt stress treatments.
**Figure S6** Proline content of mutant lines, OE lines and the WT upon the drought and salt stress treatments.
