# Supplementary material for: A breeding strategy for improving drought and salt tolerance of poplar based on CRISPR/Cas9
Source: Plant Biotechnol J. 2023 Aug 3;21(11):2160–2. doi: 10.1111/pbi.14147 (PMC10579702; doi:10.1111/pbi.14147)
Supplement: Supplementary file 1 — Figure S1 Multiple alignment, phylogenetic analysis and expression analysis of PagHyPRP1A and PagHyPRP1B. Figure S2 Healthy plants with consistent growth were selected for simulated soil drought (20% PEG‐6000) and salt (150 mM NaCl) stress tests. Figure S3 Stem diameter, stem dry weight and root‐shoot ratio of mutant lines, OE lines and the WT under drought and salt stress. Figure S4 Phenotypes analysis of mutant lines, OEs, and WT upon the stress treatment in vitro for 30 days. Figure S5 The contents of O2·− and H2O2 in mutants, OEs and WT upon the drought and salt stress treatments. Figure S6 Proline content of mutant lines, OE lines and the WT upon the drought and salt stress treatments. [file PBI-21-2160-s001.doc]

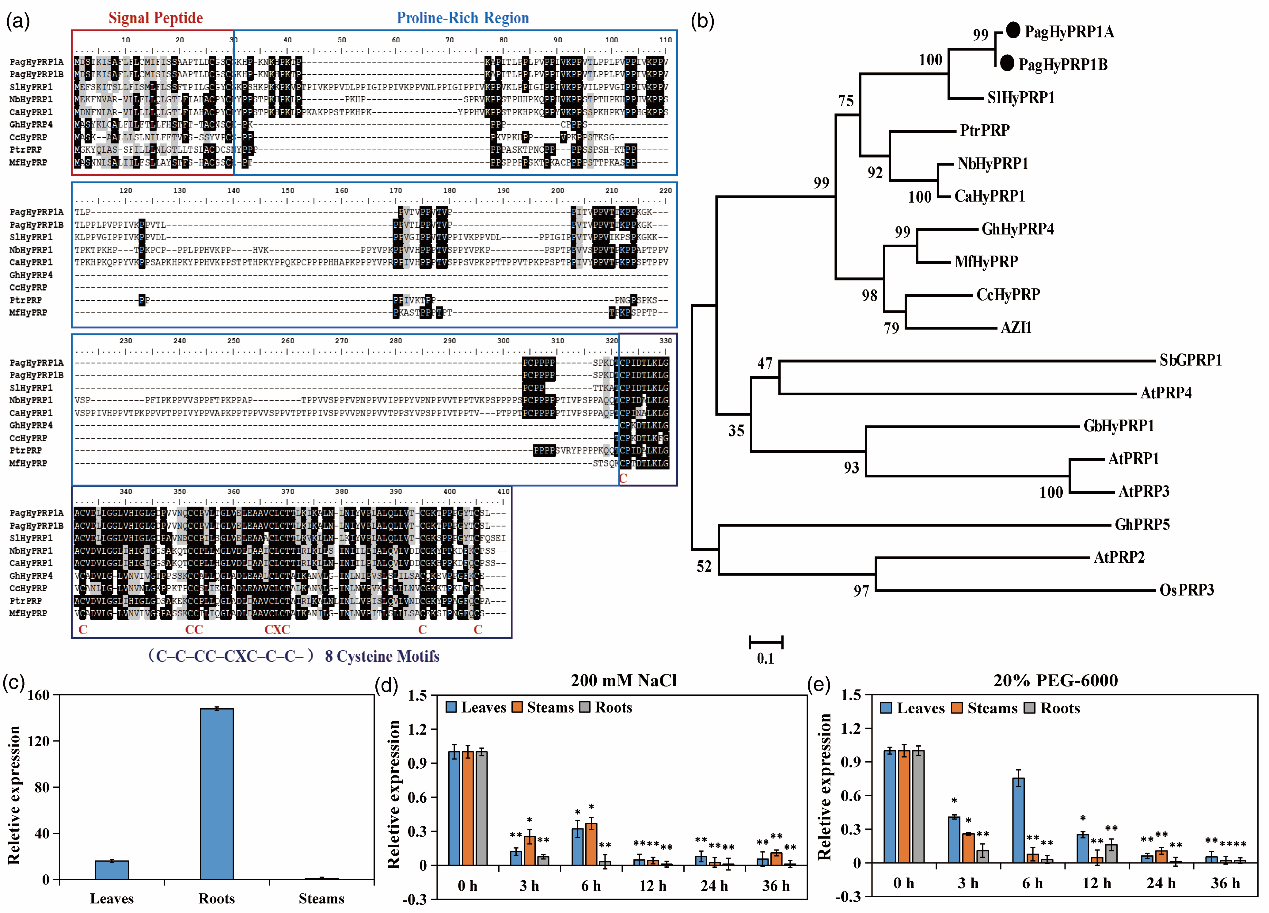


**Figure S1** Multiple alignment, phylogenetic analysis, and expression analysis of *PagHyPRP1A* and *PagHyPRP1B*. (a) Sequence alignment of the deduced PagHyPRP1A and PagHyPRP1B sequences with other HyPRP sequences. The signal peptides are framed in red, the proline-rich region is framed in light blue, and the C-terminal eight cysteine motifs are framed in dark blue. Identical amino acids are shaded in black, and similar amino acids are shaded in gray. (b) Phylogenetic tree analysis of PagHyPRP1A, PagHyPRP1B and reported HyPRP proteins from other plant species using the neighbor-joining method in the MEGA v.5.0 software. The scale bar represents 0.1 substitutions per site. (c) Relative expression level of *PagHyPRP1* in the leaf, root, and stem. The expression level in the stem was normalized to 1. (d, e) Relative expression levels of *PagHyPRP1* in leaves, roots and stems of poplars upon the 200 mM NaCl and 20% PEG-6000 stress treatments. Data = means ± SD, error bars represent standard deviation (SD) (n = 3).


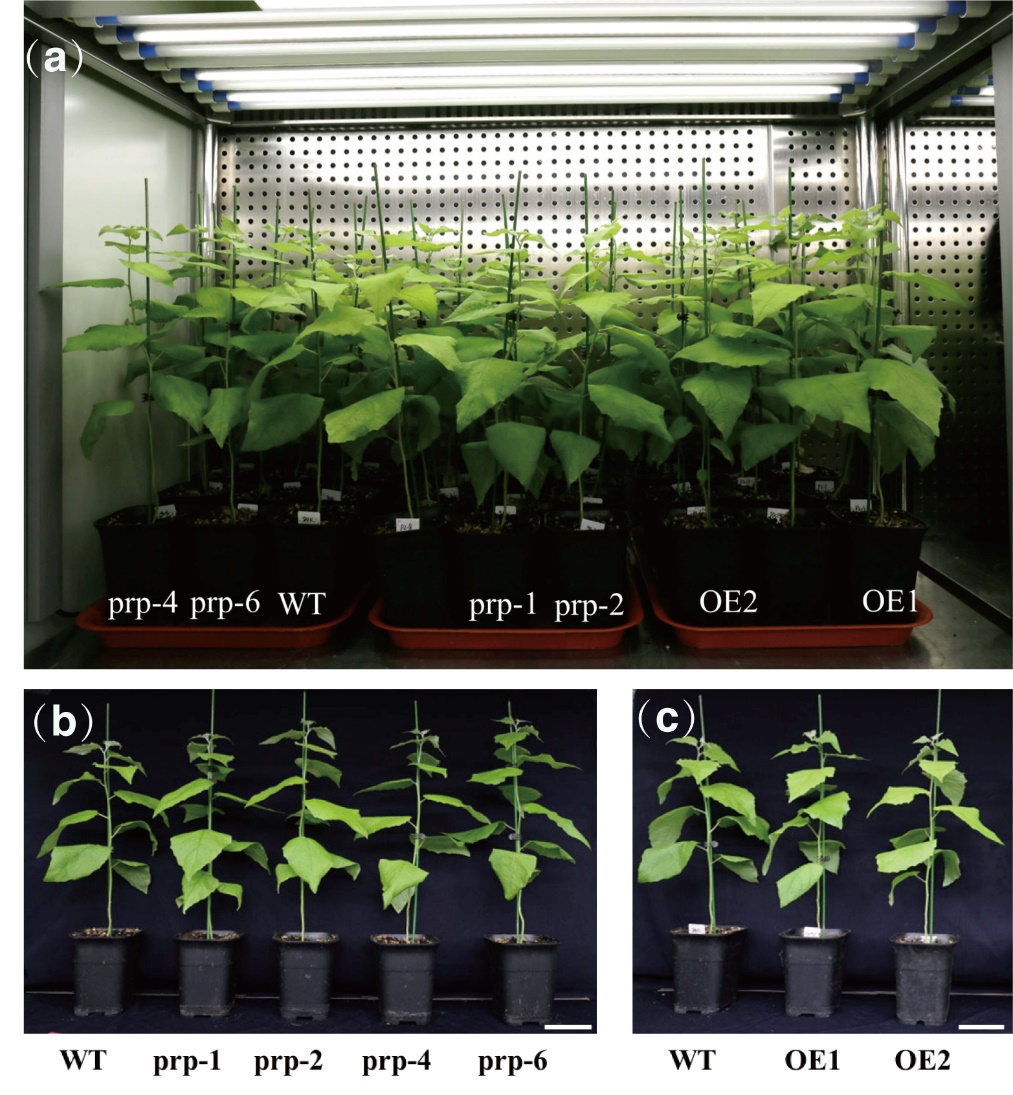


**Figure S2** Healthy plants with consistent growth were selected for simulated soil drought (20% PEG-6000) and salt (150mM NaCl) stress tests. (a) Growth status of different lines in one of the experimental groups before the stress treatment; (b) Consistent growth of Mutant lines and WT; (c) Consistent growth of OE lines with WT. Bars = 10 cm.


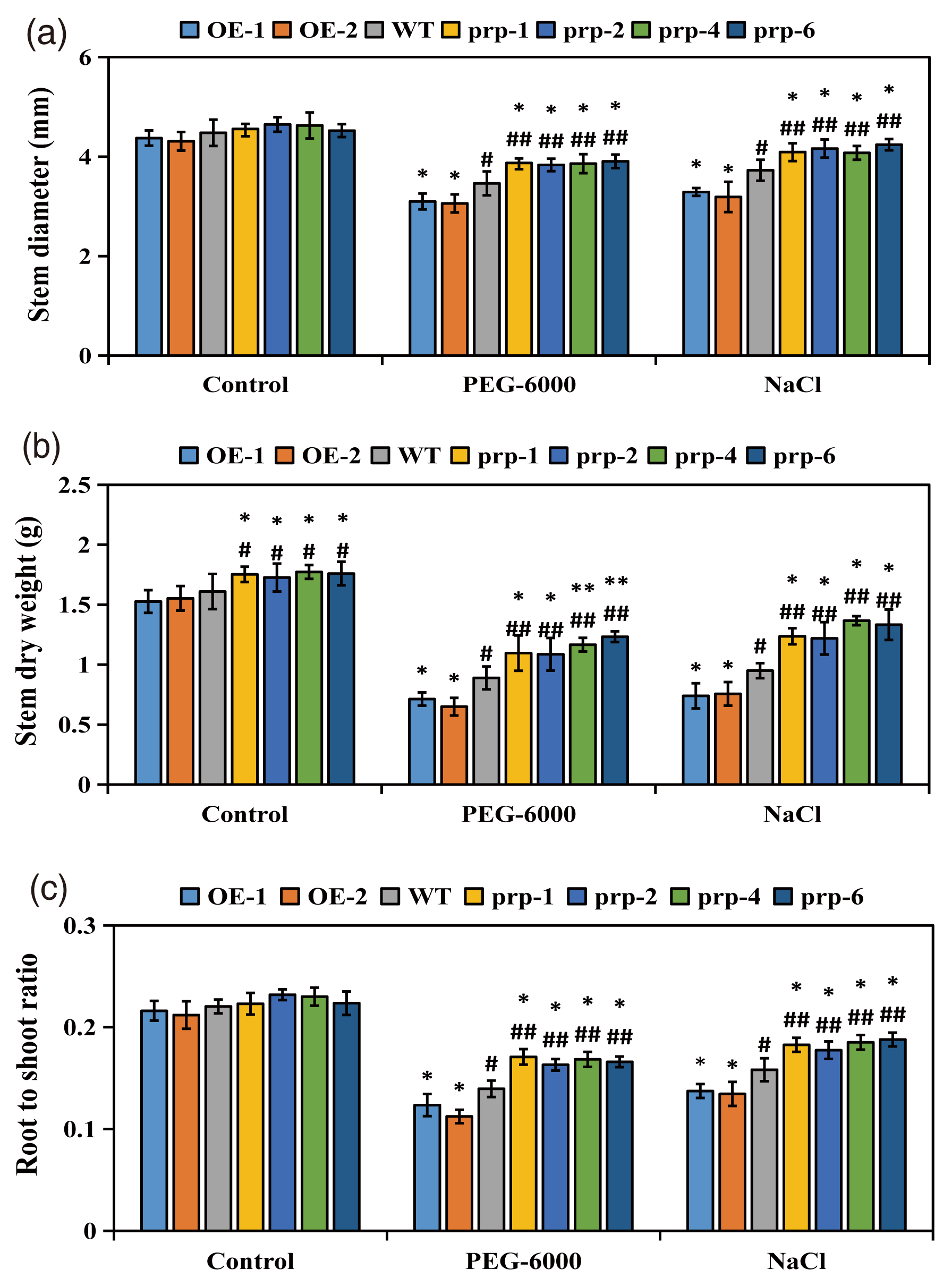


**Figure S3** Stem diameter, stem dry weight, and root-shoot ratio of mutant lines, OE lines and the WT under drought and salt stress. Data = means ± SD, error bars show standard errors (n = 6), *, ** indicates significant differences from WT (**p* < 0.05; ***p* < 0.01). #, ## indicates significant differences from OEs (#*p* < 0.05; ##*p* < 0.01).


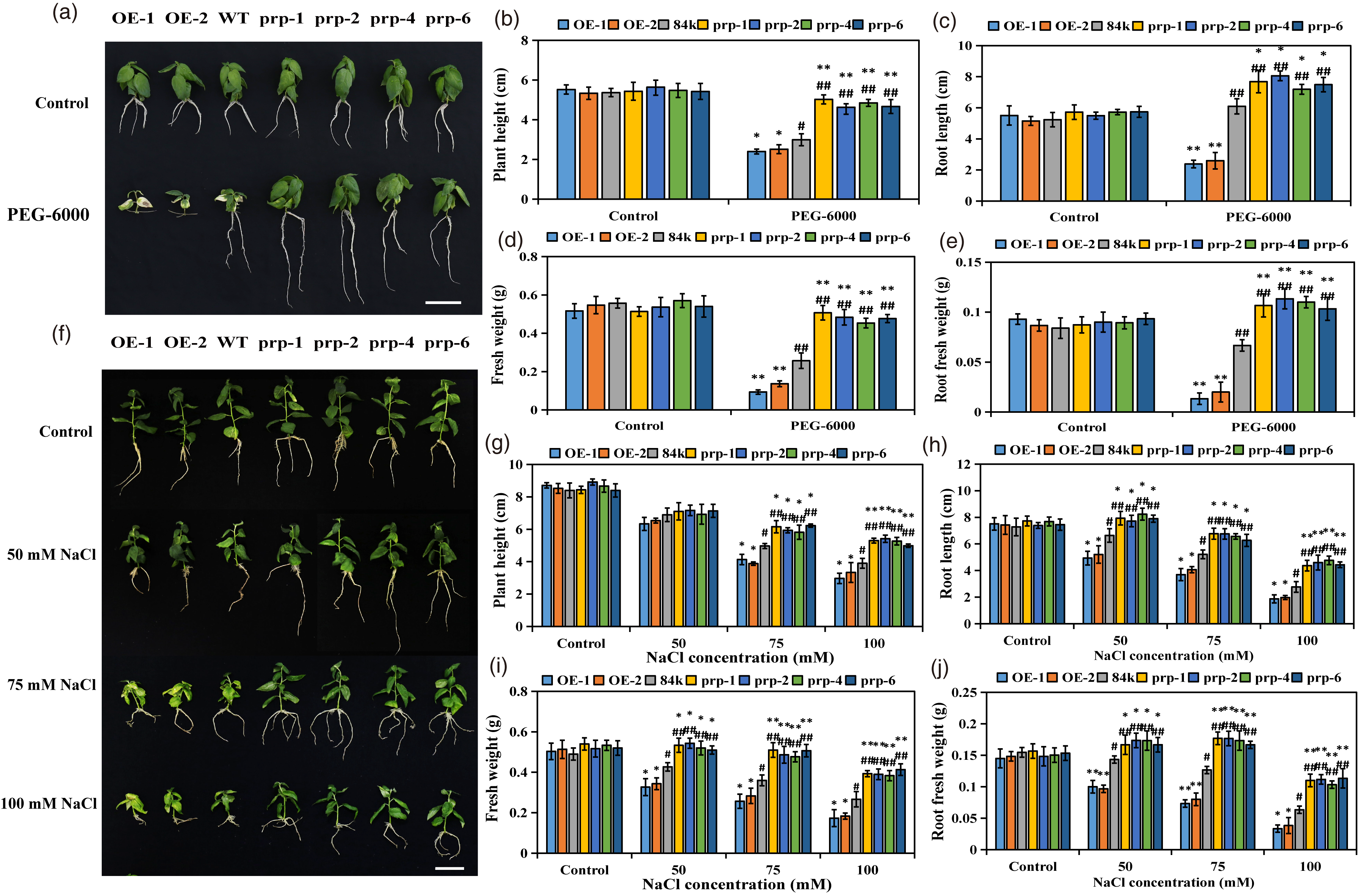


**Figure S4** Phenotypes analysis of mutant lines, OEs, and WT upon the stress treatment *in vitro* for 30 days. (a) Phenotypes analysis of mutant lines, OEs, and WT *in vitro* with 3% PEG-6000 treatment. (f) Phenotypes analysis of mutant lines, OEs, and WT *in vitro* with 0, 50, 75, and 100 mM NaCl treatment. (b-e) Plant height, root length, whole plant fresh weight, and root fresh weight of mutant lines, OEs, and WT upon the 3% PEG-6000 stress treatment. (g-j) Plant height, root length, whole plant fresh weight, and root fresh weight of mutant lines, OEs, and WT upon the NaCl stress treatment. Bars = 5 cm. The experiment was repeated three times with two plants each time. Data = means ± SD, error bars show standard errors (n = 6), *, ** indicates significant differences from WT (**p* < 0.05; ***p* < 0.01). #, ## indicates significant differences from OEs (#*p* < 0.05; ##*p*< 0.01).


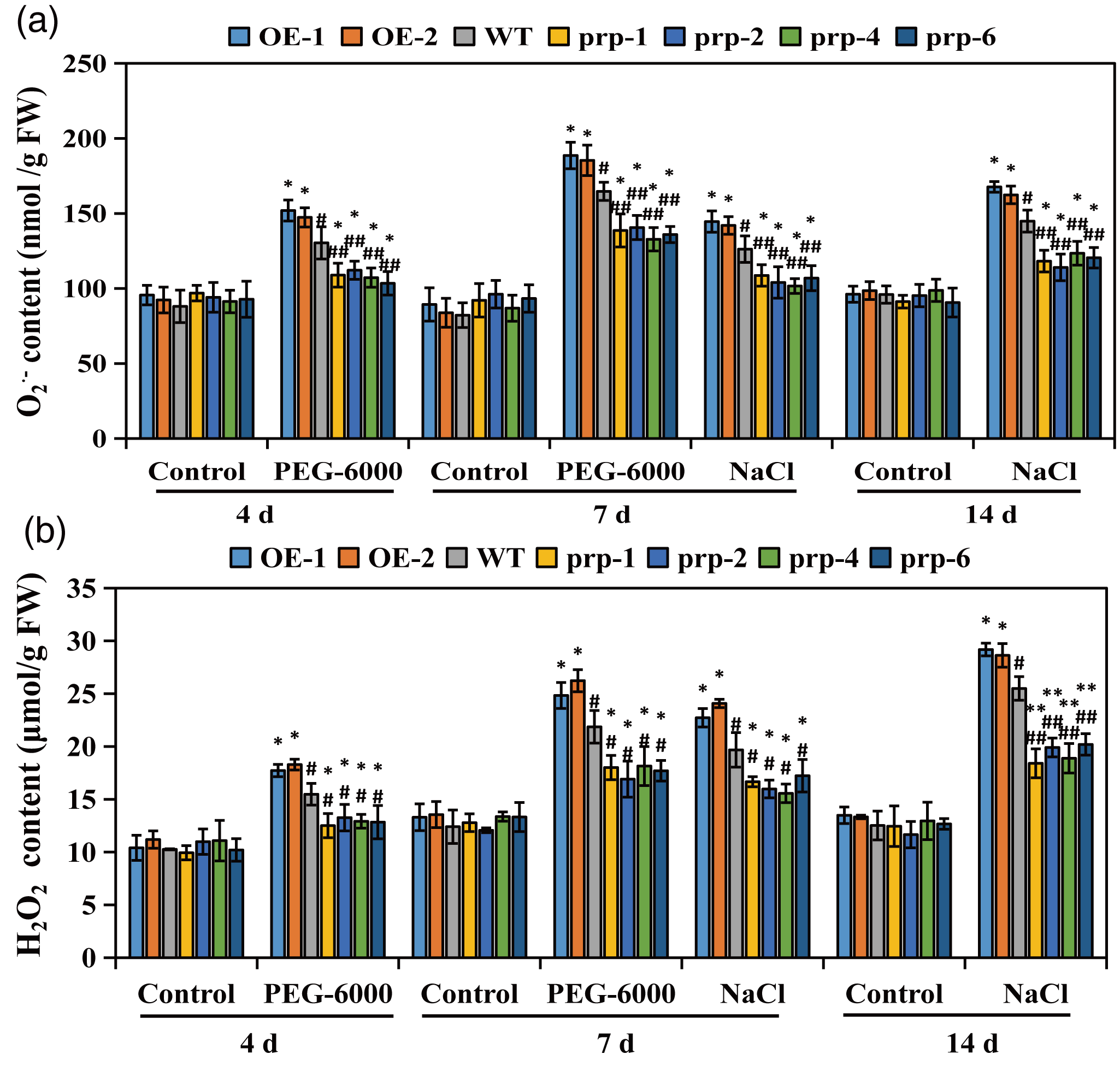


**Figure S5** The contents of O_2_^.-^ and H_2_O_2_ in mutants, OEs and WT upon the drought and salt stress treatments. Data = means ± SD, error bars show standard errors (n = 6), *, ** indicates significant from the WT (**p* < 0.05; ***p* < 0.01). #, ## indicates significant differences from OEs (#*p* < 0.05; ##*p* < 0.01).


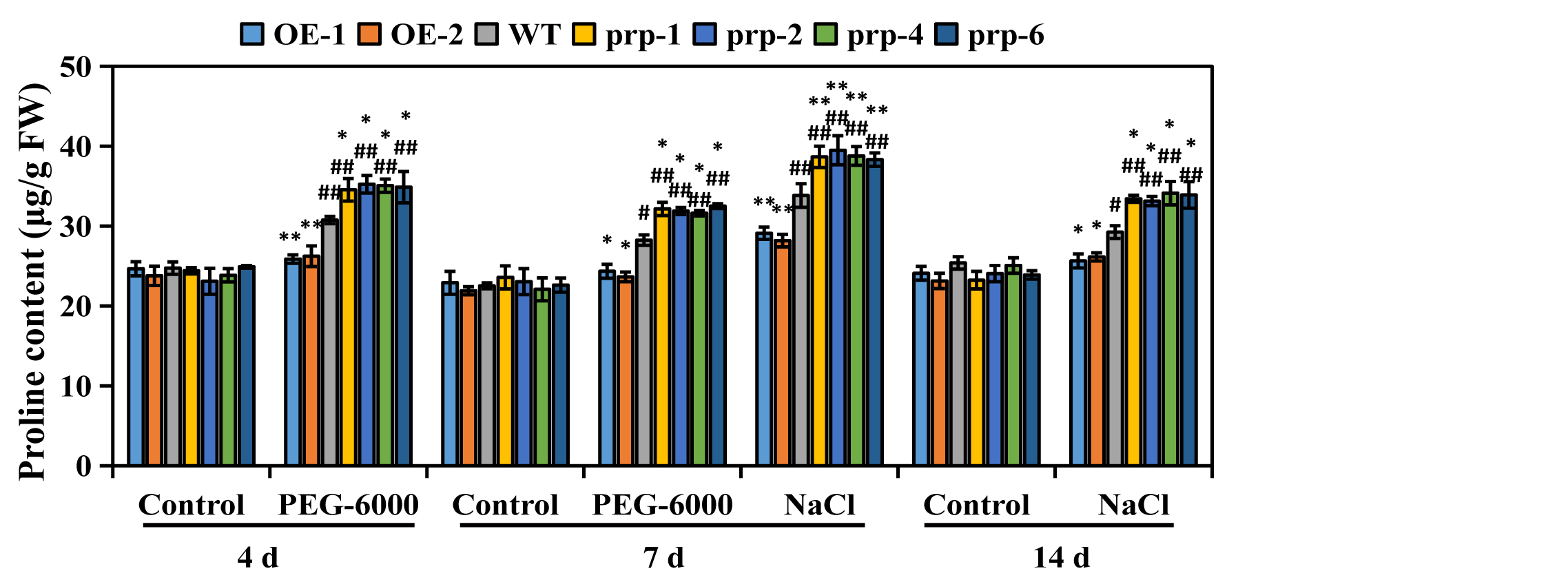


**Figure S6** Proline content of mutant lines, OE lines and the WT upon the drought and salt stress treatments. Data = means ± SD, error bars show standard errors (n = 6), *, ** indicates significant differences from WT (**p* < 0.05; ***p* < 0.01). #, ## indicates significant differences from OEs (#*p* < 0.05; ##*p* < 0.01).
